# Nutrient Addition Dramatically Accelerates Microbial Community Succession

**DOI:** 10.1371/journal.pone.0102609

**Published:** 2014-07-22

**Authors:** Joseph E. Knelman, Steven K. Schmidt, Ryan C. Lynch, John L. Darcy, Sarah C. Castle, Cory C. Cleveland, Diana R. Nemergut

**Affiliations:** 1 Institute of Arctic and Alpine Research, University of Colorado, Boulder, Colorado, United States of America; 2 Department of Ecology and Evolutionary Biology, University of Colorado, Boulder, Colorado, United States of America; 3 Department of Ecosystem and Conservation Sciences, University of Montana, Missoula, Montana, United States of America; 4 Environmental Studies Program, University of Colorado, Boulder, Colorado, United States of America; Argonne National Laboratory, United States of America

## Abstract

The ecological mechanisms driving community succession are widely debated, particularly for microorganisms. While successional soil microbial communities are known to undergo predictable changes in structure concomitant with shifts in a variety of edaphic properties, the causal mechanisms underlying these patterns are poorly understood. Thus, to specifically isolate how nutrients – important drivers of plant succession – affect soil microbial succession, we established a full factorial nitrogen (N) and phosphorus (P) fertilization plot experiment in recently deglaciated (∼3 years since exposure), unvegetated soils of the Puca Glacier forefield in Southeastern Peru. We evaluated soil properties and examined bacterial community composition in plots before and one year after fertilization. Fertilized soils were then compared to samples from three reference successional transects representing advancing stages of soil development ranging from 5 years to 85 years since exposure. We found that a single application of +NP fertilizer caused the soil bacterial community structure of the three-year old soils to most resemble the 85-year old soils after one year. Despite differences in a variety of soil edaphic properties between fertilizer plots and late successional soils, bacterial community composition of +NP plots converged with late successional communities. Thus, our work suggests a mechanism for microbial succession whereby changes in resource availability drive shifts in community composition, supporting a role for nutrient colimitation in primary succession. These results suggest that nutrients alone, independent of other edaphic factors that change with succession, act as an important control over soil microbial community development, greatly accelerating the rate of succession.

## Introduction

Deglaciated forefields have been valuable model systems for developing and testing theories of succession and have greatly enhanced our understanding of the relationship between community structure and function during ecosystem development [1]–[3]. Shifts in soil nutrient pools, including increases in available nitrogen (N) and phosphorus (P), have been well documented along early primary successional chronosequences [4]–[6] and have been shown to correlate with changes in plant community succession [1], [7], [8]. Recently, studies in such systems have revealed that – like plants – microbial communities also progress through successional stages [9]–[11]. However, the forces that control microbial succession are not well understood.

Some evidence suggests that shifts in nutrient availability may also, in part, drive microbial community succession. For example, in primary successional ecosystems, research has corroborated relationships between natural gradients in soil nutrients and microbial community composition [12], [13]. Such correlations can be difficult to interpret, however, as changes in microbial community composition could be both a cause and consequence of shifts in soil fertility. Furthermore, the mechanisms underlying correlations between standing nutrient pools and microbial communities may be temporally disconnected, in that current soil biogeochemical status may not accurately reflect the historical nutrient conditions that structured the microbial community. Thus, manipulation experiments are essential in evaluating the direct impact of nutrients and their limitations on microbial communities. Indeed, fertilizer treatments are known to elicit changes in soil microbial community structure and function in more developed ecosystems [14], [15] suggesting that nutrient availability may also be important in controlling successional changes in microbial community composition.

Yet, it would be surprising if nutrients alone drove microbial community succession for several reasons. First, other edaphic properties also undergo concomitant shifts with microbial community structure and function during succession, some of which are known to more strongly correlate with microbial community structure than nutrient pools in developed soils. For example, organic carbon (C) pools and pH, which typically show dramatic changes across primary successional chronosequences [2], are key determinants of soil microbial community composition at regional to global scales [16]–[19]. Second, soil microbial community structure can correlate with plant community composition [20], [21], which can show strong spatial gradients in early succession [3]. Third, stochastic processes can be key in shaping early successional communities where the importance of dispersal events may be accentuated, [22]–[24] and arrival order may influence assembly through priority effects [25]. Given the large functional and phylogenetic diversity of microbial communities, it is possible that succession is influenced by a diverse combination of such factors [26].

Thus, the extent to which nutrients themselves influence microbial community assembly outside of the myriad of factors that change over succession is unknown. To specifically isolate the effects of nutrients, we performed a full factorial N×P fertilization experiment in soils that had been exposed for ∼3 years in the forefield of the Puca Glacier in Southeastern Peru. We analyzed soil bacterial communities before and one year following nutrient additions and compared them with soils sampled from three different locations over an 85-year section of the Puca Glacier chronosequence. The Puca Glacier soils constitute an autotrophic successional sequence [27], and both photosynthesis and respiration respond strongly to P additions in microcosms [28], [29]. Nitrogen appears to be limiting in this system as well and N-fixation rates in 4 year old unvegetated soils are comparable to rates measured in developed soil crusts [30]. Thus, given work that demonstrates relationships between nutrients and microbial community composition, we hypothesized that fertilizer additions to early successional soils would drive communities to be compositionally different than unfertilized (control) soils. However, due to the potential influence of other edaphic (e.g. pH, organic C, soil moisture) and stochastic factors on microbial succession, we hypothesized that fertilized communities would be unique from communities found along the natural chronosequence.

## Materials and Methods

### Study site description, fertilization, and sampling

The study site is located in the forelands of the Puca Glacier in the Cordillera Vilcanota of Peru (13°46′24″S, 71°04′17″W, ∼5,000 m.a.s.l.). No specific permits were required for our field studies and our work did not involve endangered or protected species. Mean annual precipitation is roughly 100 cm and mean annual temperature is ∼5°C. Moraine rocks at this site have high quartz and calcite mineral content. Further details of this site can be found in previous work [9], [30] and soil characteristics are presented in Table S2.

We established permanent plots (1 m^2^) near the terminus of the glacier, in soils that had been deglaciated for approximately 3 years at the time of initial sampling. Corners were marked with long nails (approximately 15 cm shank length) to guide resampling. Sampling occurred in August 2010 (pre-treatment) and August 2011 (post-treatment). All of the plots were unvegetated and no mosses and lichens were present at the time of establishment. Each of the 16 plots was randomly chosen to receive one of three nutrient amendments (nitrogen addition (+N), phosphorus addition (+P), the combination of the two (+NP)) or to serve as controls, resulting in a total of four plots per treatment and four control plots.

Pre-weighed amounts of fertilizer were dissolved in glacier-melt stream water and fertilizer solutions were applied with handheld sprayers. Each sprayer was designated for a particular treatment to avoid cross contamination. For the +N plots, nitrogen was added in the form of ammonium nitrate (NH_4_NO_3_) resulting in 15 g of NH_4_NO_3_ and 5.25 g of N/m^2^. The +P plots received 0.5 g of phosphorus in the form of 2.2 g of potassium dihydrogen phosphate (KH_2_PO_4_). +NP plots received 15 g of NH_4_NO_3_ and 2.2 g of KH_2_PO_4_. For controls, stream water from the same source was sprayed onto the plots. These levels of nutrient addition were designed to result in a pulse of nutrients that would greatly overcome any possible natural limitations.

Plots were sampled prior to the application of fertilization treatment. In each plot surface soil was collected (0–5 cm) from 2 locations, and samples were composited to generate one sample per plot. Samples were obtained in the same manner one year following the fertilization treatment. Ethanol and paper towels were used to sterilize the tools before sampling each individual plot.

Samples were collected in a similar manner along three transects of varying age across the glacial forefield both years; molecular analyses were done on the samples collected in 2011. These reference soils represented advancing stages of succession: soils that had been exposed for approximately 5 years, soils with biological soil crust formation (approximately 20 years after exposure), and soils with 25–50% vegetation cover (approximately 85 years after exposure). At the field site, samples were kept in a cooler on ice for transport to Boulder, CO. Soils were sieved (to 2 mm), and then stored at 4°C for soil characterization. A subsample was immediately archived in a −80°C freezer for molecular analysis and later used for KCl extractions.

### Soil Analysis

Gravimetric soil moisture and pH (using a ratio of 2 g soil to 4 mL DI H_2_O) were assayed based on standard methods [9]. For total organic C analysis, carbonate (inorganic C) removal was first performed on dried, ground soils [9]. 50 mg of these processed soils were packed into tin capsules; %C and %N were determined using a Thermo Finnigan EA 1112 Series Flash Elemental Analyzer (Thermo Fisher Scientific, Inc., Waltham, Massachusetts, USA) [31]. Bio-available P concentrations were measured on air-dried and sieved soil (2 mm×2 mm) by extracting 3–5 g of soil with 0.5 M sodium bicarbonate for 30 minutes [32]. Extracts were filtered and analyzed colorimetrically using the ammonium molybdate-malachite green method [33] adapted for microplate analysis. NH_4_^+^ and NO_3_^−^/NO_2_^−^ extractable N were analyzed from soils using 2M KCl with 1 hour shaking and a 22 hour extraction period [34]. This analysis was performed on soils that were frozen at −80°C. Although not fresh samples, these soils typically withstand extreme fluctuations in temperature [35] and the data presented here are intended for within study comparison only. NH_4_^+^ and NO_3_^−^/NO_2_^−^ were measured on a Lachat QuikChem 8500 Flow Injection Analyzer (Lachat Instruments, Hach Company, Loveland, CO) and BioTek Synergy 2 Multidetection Microplate Reader (BioTek, Winooski, VT) respectively.

### DNA Extractions for 454 pyrosequencing

MO BIO PowerSoil™ DNA Isolation kits were used as per the manufacturer's instructions for DNA extractions of soil samples (Mo Bio Laboratories, Inc., Carlsbad, CA). PCR-amplified bacterial 16S rRNA genes from the genomic DNA of the soil samples were generated using a universal bacterial 27F and 338R primer set as described by Hamady et al. [36], and reaction conditions followed those described by Fierer et al. [37], though modified to 25 PCR cycles. Primers included a 2 bp linker, the 454 Roche Titanium A/B primer, and a unique, 12 base pair error-correcting Golay barcode for pyrosequencing as detailed by Knelman et al. [21]. 454 Life Sciences GS FLX Titanium pyrosequencing of the 16S rRNA gene amplicons was completed by the Duke Institute for Genome Sciences & Policy (Duke University, North Carolina).

### Pyrosequence and statistical analysis

Using QIIME, sequences were limited to those of a sequence length of 200 to 400 base pairs, a maximum of 5 homopolymers, a minimum quality score of 25, and a maximum of ambiguous bases/primer mismatches of 0; reverse primers were removed, and all samples were then denoised using flowgram clustering in QIIME [38]. Chloroplast sequences were removed. OTUs were selected at a 97% identity level by clustering based on representative sequences via UCLUST [39]. The Ribosomal Database Classifier [40], a naïve Bayesian classifier, was employed to assign taxonomic identification to OTUs. After sequence alignments based on the NAST algorithm [41], a phylogeny was constructed with the FastTree algorithm [42]. OTU tables were rarified to the lowest number of sequences in a sample: 407 for community dissimilarity analyses of fertilization plots. Reference transects of advancing age included 6, 5, and 3 sequenced replicate samples, respectively, and were rarefied to 71 to include all of these samples. For comparison of reference samples and fertilization plots this workflow was repeated. In order to examine differences among bacterial communities, pairwise distance matrices based on weighted UniFrac, a phylogenetic distance metric, were generated for entire communities and the cyanobacterial subset of communities in fertilization plots [43], [44]. The Principal Coordinate Analysis (PCoA) ordinations were constructed based on OTU tables and weighted UniFrac distance matrices for overall communities. The QIIME-generated OTU tables were used to evaluate the relative abundance of all taxa.

Primer v6 software [45] was used to perform permutational ANOVAs (PERMANOVA) to compare phylogenetic distances among bacterial communities. PERMANOVA tests were used on both UniFrac beta diversity matrices of the entire communities and cyanobacterial portions of communities. PERMANOVA analysis was also employed to assess differences among treatment-affected communities and successional reference communities. For all comparisons with reference communities, data were rarefied to the lowest sampling depth among both fertilization plot and reference plot samples.

R software [46] was used for further statistical analysis. The PERMDISP2 procedure (with permutational P-values) from the R vegan package to test homogeneity of group dispersions (variances) was also employed via QIIME in order to test for differences in community phylogenetic dispersion (UniFrac) in fertilized samples and reference successional communities [47], [48]. As well, the pgirmess package in R was used to evaluate comparisons among reference chronosequence soil relative abundance data via the Kruskal Wallis test. To assess treatment vs. temporal effects underlying shifts in overall phylogenetic community composition, a Tukey's HSD post-hoc test was used to compare UniFrac distances of paired pre- and post-treatment plots with paired control plots from both years. Additionally, to assess the relative abundances of bacterial taxa, we compared the differences in paired pre- to post-treatment taxon relative abundances for each treatment with that of paired control plots via Tukey's HSD post-hoc tests. To examine the relationship between treatment-related community shifts from our fertilization experiment and reference communities across advancing stages of soil development, we examined the relationship between weighted UniFrac phylogenetic dissimilarity and time between +NP communities and reference communities via a Spearman correlation Mantel test. The Mantel test tests the null hypothesis that there is no correlation between +NP and reference community dissimilarity and chronosequence age rank.

All relative abundance data and environmental variables were evaluated for normality. Taxon relative abundances and fertilizer plot NO_3_^−^/NO_2_^−^ were square root transformed to achieve a normal distribution prior to statistical analysis. All other edaphic factors were natural log transformed. ANOVAs, Tukey's HSD, and Kruskal Wallis post-hoc tests were used to assess differences in pH, %C, P, N pools and soil moisture in fertilization plots and reference chronosequence soils. Percent N was below the detection limit in a majority of samples and thus removed from statistical evaluations.

Sequences and metadata have been deposited in FigShare and are available with the DOIs: 10.6084/m9.figshare.1050042 (metadata) and 10.6084/m9.figshare.1048992 (sequences).

## Results and Discussion

**Figure 1:**
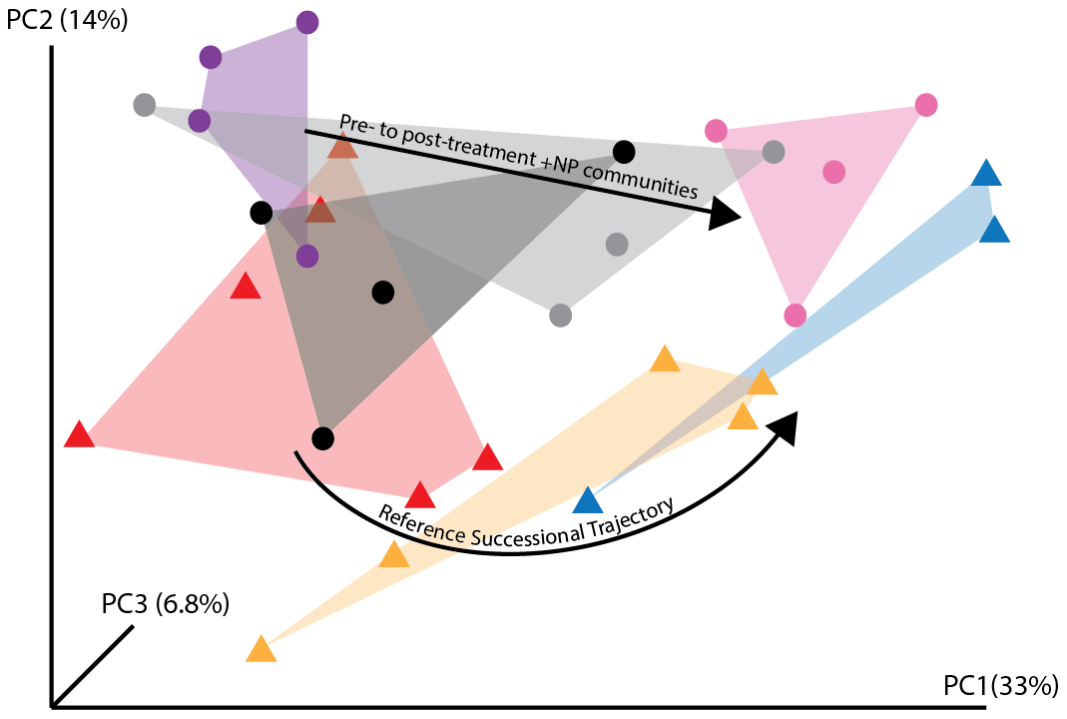
Principal Coordinates Analysis (PCoA) ordination plot of bacterial communities from the field fertilization experiment and bacterial communities from the successional chronosequence. Only the +NP treatment communities are shown because the +N and +P treatments did not result in significant community shifts. PCoA visually represents differences among community composition as the distance between points. Triangles represent communities from the natural chronosequence: red  = 5 years old; orange  = 20 years old; blue  = 85 years old. Circles represent communities from the fertilization experiment: black  =  pre-treatment control; grey  =  post-treatment control; purple  =  pretreatment +NP; Pink  =  post-treatment +NP. Our analysis revealed significant community shifts over the reference chronosequence (triangles) as well as a significant response to +NP fertilization (circles). As well, the PCoA analysis demonstrates that the +NP communities (pink circles) group with the oldest soils from the chronosequence (blue triangles).

**Figure 2:**
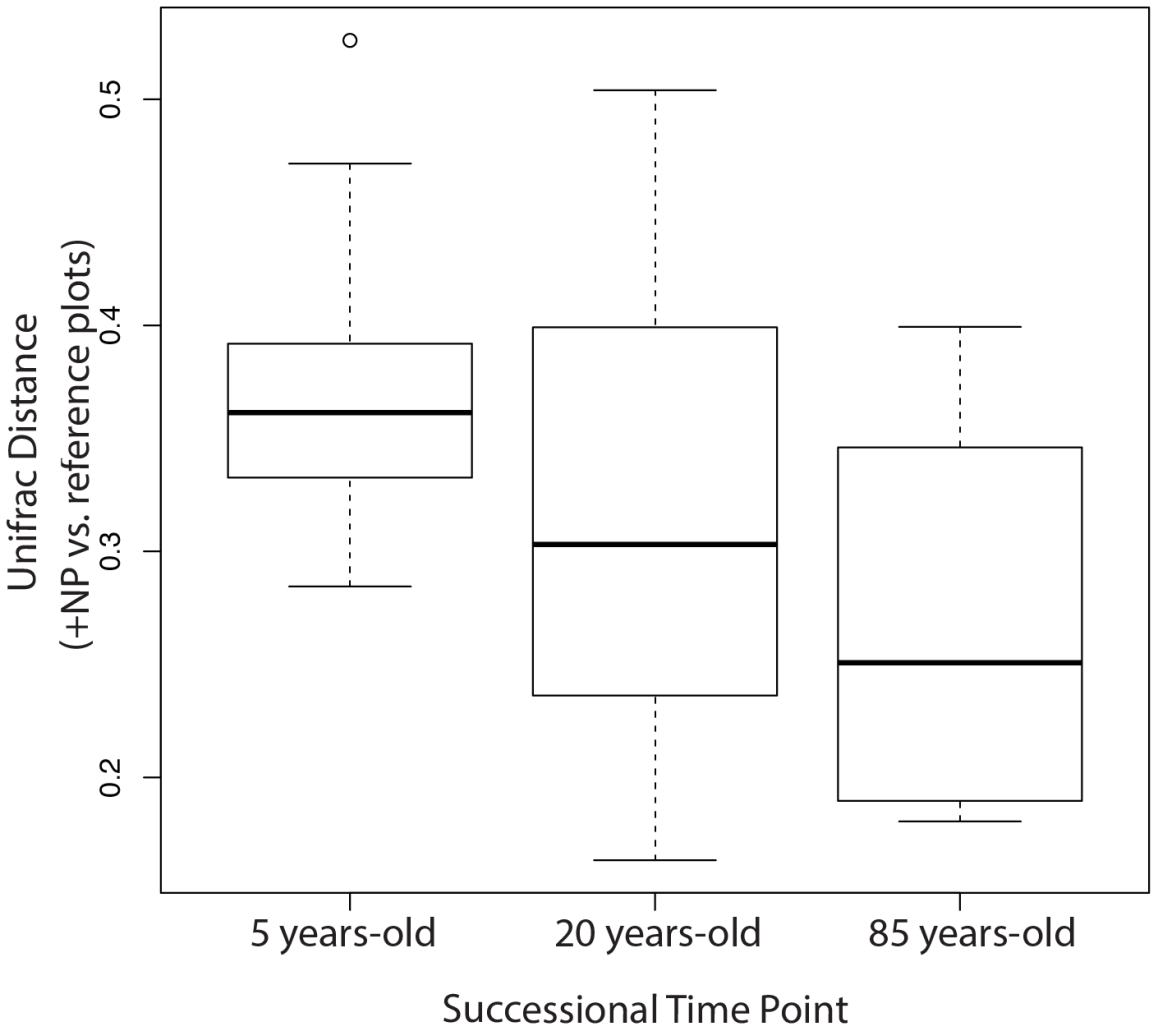
Relationship between +NP treatment-affected communities and reference communities. A box plot shows the average weighted UniFrac [43], [44] distance between +NP-treated communities and reference communities with increasing successional time. A Mantel test demonstrates that +NP communities show decreasing dissimiliarty as compared to the reference communities over advancing stages of succession (ρ_M_ = −0.35 P = 0.01).

Together, our analyses demonstrate that a single +NP application caused the bacterial community structure of the 3-year-old barren soils to converge with the structure of 85-year-old vegetated soils after only one year. First, paired pre- and post-treatment plot community differences (weighted UniFrac distance) were assessed among all plot categories using an ANOVA. The +NP plots showed a significant community shift in response to the treatment (Tukey's HSD; P = 0.037); no other significant differences in community structure were detected between treatments and controls (Tukey's HSD; P>0.05). A PCoA ordination (Fig. 1) revealed a successional trend in community composition across the reference chronosequence, with post-treatment +NP communities clustering with the oldest reference communities. A PERMANOVA analysis demonstrated that there were no significant differences among pre-treatment communities (Table 1). However, communities in post-treatment +NP plots were significantly different from both pre- and post-treatment controls, including the paired pre-treatment +NP plots (PERMANOVA, P<0.05, Table 1). When +NP communities were compared to reference communities across the natural chronosequence, a Mantel test of pairwise average UniFrac [43], [44] distances between +NP plots and reference samples revealed significant patterns of decreasing dissimilarity: +NP communities were most similar to the 85 year old successional soils (Fig. 2, ρ_M_ = −0.35 P = 0.01). The PERMANOVA analysis also showed that +NP communities were significantly different than communities of all successional stages except those of the oldest transect (85 years old) (Table 1). These results suggest that fertilization drives community composition away from early successional stages and results in convergence with communities of older soils. Likewise, the phylogenetic dispersion [47], [48] of +NP communities was significantly different from all reference communities except those in the 85 year old soils (Table 2). We note that our PERMANOVA analysis was not corrected for multiple comparisons due to the low statistical power of our study, but the general results of this analysis were nonetheless corroborated by our other statistical analyses of treatment effect (ANOVA/Tukey's HSD of pre- and post-treatment community shifts) and convergence of the +NP plots to the oldest successional soils (Mantel test of +NP community distance compared to successional reference samples over time).

**Table 1:** Post-treatment +NP phylogenetic community structure was significantly different from controls and from all communities from the reference chronosequence with the exception of communities in the oldest soils (P<0.05).

Permutational MANOVA (PERMANOVA) contrast P-values				
Sample vs. Sample	post-treatment control	post-treatment +N	post-treatment +P	post-treatment +NP
pre-treatment control	0.415	0.066	**0.031**	**0.026**
post-treatment control	---	0.422	0.072	**0.036**
pre-treatment +NP-paired	0.114	0.085	**0.024**	**0.032**
post-treatment +NP plots	**0.036**	**0.023**	0.18	**---**
succession timepoint 1	0.124	**0.003**	**0.005**	**0.006**
succession timepoint 2	0.105	**0.009**	**0.018**	**0.022**
succession timepoint 3	0.152	0.055	0.179	0.162
Significant P-values (P<0.05) bolded.				

Controls showed no differences from any contrasts (P>0.05). Significant P-values (P<0.05) are bolded.

**Table 2:** Post-treatment +NP communities showed differences from all reference succession communities with the exception of the oldest transect (P<0.05).

Homogeneity of Dispersion (PERMDISP) P-values		
Sample vs. Sample	post-treatment +NP	post-treatment control
succession timepoint 1	**0.022**	0.508
succession timepoint 2	**0.042**	0.588
succession timepoint 3	0.555	0.997
Significant P-values (P<0.05) bolded.		

Controls showed no difference in community dispersion from communities of any of the reference succession transects (P>0.05). Significant P-values (P<0.05) are bolded.

Our results suggest that nutrient colimitation is an important control on microbial primary succession in this system. Because of low statistical power, it is difficult to discern whether this colimitation is simultaneous, meaning that both nutrients need to be present for a community response, or independent, meaning that each nutrient in isolation may elicit some response [49]. However, there is some evidence that single nutrient additions may cause a smaller response than when both nutrients are abundant. For example, our results show that post-treatment +P communities are not significantly different from post treatment +NP communities (Table 1). As well, both +N and +P plots show patterns of convergence similar to +NP plots in comparison with ongoing natural succession; by contrast, control plots do not display convergence (Table 1). Thus, +N and +P communities may represent intermediate states between control and +NP plots, but we were not able to statistically demonstrate an underlying treatment effect.

While our study is unique as we established and resampled nutrient addition plots in a remote glacial forefield, the rapidly changing nature of the Puca Glacier landscape and criteria for setting up plots on a stable and relatively homogenous surface limited replication and necessitated rarefaction of sequencing depth to 71 to include all available samples. As such, we acknowledge the need to be circumspect in drawing conclusions as such factors curbed the statistical power of our study and potentially our ability to detect smaller magnitude treatment effects in the +N and +P additions, for example. However, we note that the patterns shown here are robust to even lower rarefaction depths (55–70); thus, it is likely that observed patterns are real. Nonetheless, our research shows the greatest, and only statistically significant treatment effect on microbial communities under +NP additions, suggesting the effect of both nutrients in tandem is important in succession.

Interestingly, standing nutrient pool analysis lends some insight into particular dynamics that may underlie nutrient colimitation in this autotrophic chronosequence. For example, +P and +NP soils both show significant increases in ammonium pools in comparison with control plot soils (Table S1), which is consistent with a body of research that demonstrates P limitation is a strong control of N-fixation [50], [51], and may be particularly strong in this autotrophic chronosequence that features cyanobacterial N-fixers [9]. Likewise, +N plots show a significant increase in bioavailable-P relative to control plots (Table S1), a pattern supported by research that shows N availability may limit the production of phosphatase enzymes [51]–[53]. Thus, these particular biochemical pathways lead to a coupling of nutrient cycles, which appears to be reflected in a colimitation to successional processes.

Despite the multitude of well documented changes across successional gradients including shifts in pH, C pools, plant cover and biotic historical factors, nutrient addition alone not only caused changes in early successional community structure, but induced convergence with late successional soil communities of the natural chronosequence (Fig. 1 and 2 and Tables 1 and 2). For example, strong changes in %C, another known filter on microbial communities, were observed across the natural chronosequence but not in +NP plots (Tables S1 and S2). In other ecosystems, the effects of fertilization on microbial community structure have been attributed to changes in plant productivity or community structure [14]. However, it is important to note that while the +NP fertilization caused sparse vegetation (<15 cm tall) to colonize after one year at our site, soils were collected at least 75 cm from these small plants. Altogether, our results suggest that the effects of the +NP fertilization on microbial community succession were direct and not mediated through changes in other aspects of the abiotic environment or through the effects of plants on soil communities.

Our field-based fertilization experiment helps to extend existing ecological theory regarding the role of nutrient limitations in succession [4], [5], [54] to microbial communities present in the earliest primary successional soils, which are important for biogeochemical cycling, physical soil development, and plant colonization [9], [21], [30]. While it is widely acknowledged that microbes can alter soil fertility and nutrient cycling processes, and that changes in soil nutrient pools and microbial communities occur over primary succession [9], [12], [13], [30], to what extent nutrients directly structure soil microbial communities is not clear. Our fertilization experiment allowed us to decouple the effects of changes in microbial communities on nutrient cycles and to directly demonstrate the influence of nutrient pools on microbial succession. Correlative studies are less powerful because they cannot isolate the impact of individual factors amidst the multiplicity of soil properties that change with succession, and because measured soil properties may be decoupled from microbial community composition in time.

Despite the high fertilization rate we used, the nutrient addition treatment did not push communities to an alternative or novel state, but simply accelerated succession, rapidly producing a community that was structurally most similar to the community in the 85 year old soils in the chronosequence (Fig. 1 and 2 and Tables 1 and 2). Thus, our data highlight the stability of soil microbial communities [55]. Few studies have explicitly evaluated nutrients in the context of longer-term successional reference plant communities to understand how nutrients may either drive succession or shape alternative stable states in communities. However, in a study of salt marsh vegetation, Van Wijnen and Bakker [56] observed that fertilization of young marsh communities resulted in plant communities that resembled those of older, unfertilized marshes. These results further suggest that nutrient-related mechanisms for succession may be generalizable between plant and microbial communities.

The relative abundance of cyanobacteria significantly increased in the +NP plots and the phylogenetic structure of the cyanobacterial communities in post-treatment +NP plots was significantly different from the paired pre-treatment +NP and pre-/post-treatment control plots (PERMANOVA, P<0.05, Table S3). Although not significant, cyanobacterial relative abundance nearly doubled between the oldest and youngest stages of the reference chronosequence and past work at this site has documented similar successional changes in cyanobacterial community structure (Table S2) [9], [30]. Consistent with these results, a laboratory experiment evaluating microbial autotrophs from this site demonstrated that P additions resulted in significant increases in the growth rate of photoautotrophic crusts [28]. Both N fixation rates and the relative abundance of N-fixing cyanobacteria show successional trends at this site as well [30], suggesting that N availability may also limit microbial growth and activity. The current study adds to this work and demonstrates that both N and P together are important colimiting controls over community successional processes in this system (Tables 1 and 2).

The increase in the relative abundance of cyanobacteria in the +NP plots may reflect their ecological advantage in this low C environment. In a laboratory study, Drakare [57] observed that P additions enhanced cyanobacterial populations, but only in an environment where low C concentrations constrained heterotrophic growth. Incubation studies of early successional soils that found increases in heterotrophic activity in response to both N and C (but not to N alone) are also consistent with this interpretation [58], [59]. These results indicate that C often limits the response of the heterotrophic community to nutrient additions, whereas cyanobacteria can readily take advantage of such nutrients to fuel photosynthesis. By extension, we argue that the observed effects of N and P additions on microbial community succession are likely to apply only to autotrophic successional sequences, and that heterotrophic succession (sensu Fierer et. al [27]) may be controlled by a different suite of resources, including C availability.

Microbes are fundamental to soil physical and chemical development and underlie ecosystem function, thus understanding the factors that drive soil microbial community succession is key to predicting and managing ecosystem development. Particularly in low nutrient environments, microbial activity has major effects on soil, plant community, and ecosystem development [9], [30], [60], [61]. Likewise, low nutrient environments may feature more prominent nutrient colimitations [49]. As such, the results of this study have important implications for understanding nutrient controls on ecosystem development and relevant models for microbial succession. Furthermore, while early successional microbial communities may vary strongly in both composition and in terms of the specifics of resource availability (e.g., heterotrophic vs. autotrophic), our study provides evidence that nutrient colimitation may provide a generalizable mechanism for microbial community succession in autotrophic successional sequences. Our data also support recent evidence for the stability of soil microbial communities, as fertilization simply accelerated succession and did not push communities into a novel state. Overall, the details of microbial nutrient limitations presented herein are essential to understanding the factors that structure early successional microbial communities, the profound contributions they make to soil development, and the ecosystem processes they mediate.

## Supporting Information

Table S1 **Mean of Edaphic Properties and Tukey's HSD Comparisons for Post-Treatment Plots.**
(DOCX)

Table S2 **Mean of Edaphic Properties and Cyanobacterial Relative Abundance for Reference Chronosequence.**
(DOCX)

Table S3 **Mean Relative Abundances of Major Taxa at Puca Glacier Site.**
(DOCX)
