# Supplementary material for: Nutrient Addition Dramatically Accelerates Microbial Community Succession
Source: PLoS One. 2014 Jul 22;9(7):e102609. doi: 10.1371/journal.pone.0102609 (PMC4106831; doi:10.1371/journal.pone.0102609)
Supplement: Table S1 — Mean of Edaphic Properties and Tukey's HSD Comparisons for Post-Treatment Plots. (DOCX) [file pone.0102609.s001.docx]

Table S1.
