# Supplementary material for: Nutrient Addition Dramatically Accelerates Microbial Community Succession
Source: PLoS One. 2014 Jul 22;9(7):e102609. doi: 10.1371/journal.pone.0102609 (PMC4106831; doi:10.1371/journal.pone.0102609)
Supplement: Table S2 — Mean of Edaphic Properties and Cyanobacterial Relative Abundance for Reference Chronosequence. (DOCX) [file pone.0102609.s002.docx]

Table S2.
